# Notch-Inflammation Networks in Regulation of Breast Cancer Progression

**DOI:** 10.3390/cells9071576

**Published:** 2020-06-28

**Authors:** Yulia Liubomirski, Adit Ben-Baruch

**Affiliations:** School of Molecular Cell Biology and Biotechnology, George S. Wise Faculty of Life Sciences, Tel Aviv University, 6997801 Tel Aviv, Israel; yulialiu@post.tau.ac.il

**Keywords:** breast cancer, inflammatory cells, interleukin 6, macrophages, NF-κB, Notch ligands, Notch receptors, pro-inflammatory cytokines

## Abstract

Members of the Notch family and chronic inflammation were each separately demonstrated to have prominent malignancy-supporting roles in breast cancer. Recent investigations indicate that bi-directional interactions that exist between these two pathways promote the malignancy phenotype of breast tumor cells and of their tumor microenvironment. In this review article, we demonstrate the importance of Notch-inflammation interplays in malignancy by describing three key networks that act in breast cancer and their impacts on functions that contribute to disease progression: (1) Cross-talks of the Notch pathway with myeloid cells that are important players in cancer-related inflammation, focusing mainly on macrophages; (2) Cross-talks of the Notch pathway with pro-inflammatory factors, exemplified mainly by Notch interactions with interleukin 6 and its downstream pathways (STAT3); (3) Cross-talks of the Notch pathway with typical inflammatory transcription factors, primarily NF-κB. These three networks enhance tumor-promoting functions in different breast tumor subtypes and act in reciprocal manners, whereby Notch family members activate inflammatory elements and vice versa. These characteristics illustrate the fundamental roles played by Notch-inflammation interactions in elevating breast cancer progression and propose that joint targeting of both pathways together may provide more effective and less toxic treatment approaches in this disease.

## 1. Introduction

The Notch pathway controls many developmental processes, where it dictates cell fate determination, differentiation and tissue homeostasis (representative review articles: [1,2,3,4,5,6]). Members of the Notch pathway—namely the transmembrane receptors Notch 1–4 and the transmembrane ligands Delta-like (DLL) 1, 3, 4 and Jagged (Jag) 1, 2 in mammals—also regulate pathological conditions; by mediating cell-to-cell contacts between the cancer cells themselves, and between tumor cells and adjacent cells, they control tumor growth and metastasis. Extensive research has demonstrated that the interactions between Notch receptors and ligands regulate gene transcription and intracellular events in cancer cells and in cells of the tumor microenvironment (TME), by that greatly contributing to the complex net of interactions that shapes the consequences of the malignancy process [7,8,9,10,11].

Breast cancer (BC) is one of the cancer types in which Notch signaling leads to multiple pro-metastatic events that can take place in the tumor cells themselves as well as at the TME, as has been summarized by recent reviews (e.g., [11,12,13,14,15,16,17,18]). Members of the Notch family have been extensively studied in BC, where the disease is now molecularly categorized to four main subtypes based on the expression of estrogen receptors (ERs), progesterone receptors (PRs) and human epidermal growth factor receptor 2 (HER2). The highly aggressive triple negative (TNBC) subtype is so named because it lacks the presence of these three receptors, and accordingly it cannot be treated by receptor-targeting therapies; rather, the conventional treatment in TNBC is chemotherapy. Alongside with TNBC (corresponding to the term “basal-like” in genomic analyses), the other three BC subtypes consist of luminal-A tumors that account for over 40% of the patients, express ERs/PRs only and have a relatively good prognosis; luminal-B tumors that express ERs/PRs but can also carry HER2 amplification or relatively high ki67 levels; and HER2+ tumors that lack ERs and PRs [18,19,20].

Increasing evidence indicates that Notch signaling is strongly involved in BC, generally promoting malignancy cascades [11,12,13,14,15,16,17,18]. The initial evidence for the pro-tumor roles of Notch family members in BC progression arose from mouse mammary tumor virus (MMTV) studies, where the insertion site for MMTV was Notch4, converting mammary epithelial cells to neoplastic cells in mice [21,22]. With time, it was demonstrated that many Notch family members promote pathogenesis in BC at many different stages of disease. This is illustrated for example by studies on tumor initiation (Notch1; Notch3), stem cell control (Notch1; Notch4; Jag1; Jag2; DLL1), angiogenesis (Notch1; DLL4) invasion and metastasis in remote organs (Notch1; Notch2; Jag1; DLL1) [23,24,25,26,27,28,29,30,31,32,33,34,35,36,37,38,39].

Particularly, Notch1 has emerged as an important regulator of BC progression. Alongside with findings demonstrating that Notch1 (and Jag1) expression were significantly associated with poor overall survival in BC in general [32], many studies connected Notch1 to TNBC in particular. Notch1 was found to be hyper-activated or over-expressed in TNBC, and its elevated levels were linked to poor overall survival and chemotherapy resistance [29,30,31,32,33]. Often, constitutive activation of Notch1 in TNBC resulted from gene rearrangements, deletions and mutations in the PEST domain [40,41,42]. However, in view of the fact that Notch1 activating mutations were observed in only a subset of TNBC patients [40,41,42] the identity of other regulatory mechanisms that affect Notch activities in TNBC and in BC in general, have been the subject of growing interest.

Between others, the search for defined mechanisms that control Notch activities in cancer has addressed Notch-TME interactions. Specifically, inflammatory processes were addressed in view of their fundamental roles in promoting tumor cell proliferation and invasion, immune suppression and angiogenesis [43,44,45,46,47]. Along these lines, the different studies investigated the connections between the Notch pathway and inflammatory elements: cells, soluble mediators and transcription factors. This is also so in BC, where inflammatory processes are tightly connected to more aggressive disease and chemoresistance [48,49,50,51,52,53,54,55,56,57,58,59,60,61,62].

In this review article, we exemplify the importance of Notch-inflammation networks in regulating cancer progression by focusing on BC, where both Notch and inflammation are central determinants. To illustrate the current knowledge and emphasize key processes, this manuscript focuses on three exemplary networks, in which Notch family members promote BC aggressiveness through interactions with pro-inflammatory elements: (1) Cross-talks of the Notch pathway with myeloid cells that contribute to cancer-related inflammation, specifically with macrophages; (2) Cross-talks of the Notch pathway with pro-inflammatory soluble mediators, particularly interleukin 6 (IL-6) and IL-6-driven pathways (through the transcription factor signal transducer and activator of transcription 3, STAT3); (3) Cross-talks of the Notch pathway with transcription factors that promote inflammatory processes, mainly nuclear factor-kappaB (NF-κB). Following these three sections, we conclude this article by discussing the implications of Notch-inflammation interplays in cancer research and therapy, in BC and in general.

## 2. Network No. 1: Cross-Talks of the Notch Pathway with Myeloid Cells that Contribute to Cancer-Related Inflammation

The TME in BC is characterized by the presence of myeloid cells that generally enhance tumor progression. Myeloid-derived suppressor cells (MDSCs) and N2 neutrophils are known to elevate malignancy cascades [63,64,65,66], yet with relatively limited understanding of their cross-talks with Notch family members in BC (e.g., [67]). In parallel, the macrophage sub-population is not only highly present in breast tumors but also integrates many pro-tumor activities via interactions with the Notch pathway (as described further below).

Macrophages are major contributors to cancer-related inflammation and regulate many stages of tumor progression [68,69,70]. To follow up on the conventional categorization of M1 “classically activated” and M2 “alternatively activated” macrophages, very often tumor-associated macrophages (TAMs) are considered as M2-polarized cells that promote tumor cell proliferation, angiogenesis, immune suppression and other key tumor-supporting events [70,71,72]. In parallel, M1 macrophages are generally regarded as anti-tumor cells that constitute a major source for pro-inflammatory cytokines such as tumor necrosis factor α (TNFα) and interleukin-1β (IL-1β) [70,73]; whereas these cytokines can promote anti-tumor activities at certain stages of the malignancy process, they are also often linked to chronic inflammation and to pro-metastatic effects in BC and other malignancies as well [45,74,75,76,77,78,79]. In this context, it is important to note that actually, macrophage types are not dichotomic but rather these cells demonstrate a very high level of plasticity that leads to a continuum of phenotypes and activities [80,81].

In view of their prominent roles in breast malignancy and ability to regulate cancer progression through interactions with the Notch pathway [82], Notch-macrophage cross-talks in BC exemplify well the way such networks may act to promote disease course. In this respect, it is interesting to note that a recent publication demonstrated that macrophage infiltration was denser and Jag1 expression levels were higher in BC patients who developed resistance to treatment with aromatase inhibitors (AI) than in AI-sensitive patients. In the total cohort, both parameters were significantly associated with clinico-pathological parameters and poor survival [83]. Similarly, analyses of biopsies of patients diagnosed with invasive micropapillarly carcinoma of the breast (IMPC) demonstrated significant correlation of Jag1 expression with macrophage infiltration, and both parameters were negatively linked to disease-free survival in this relatively aggressive type of disease [84].

**Figure 1:**
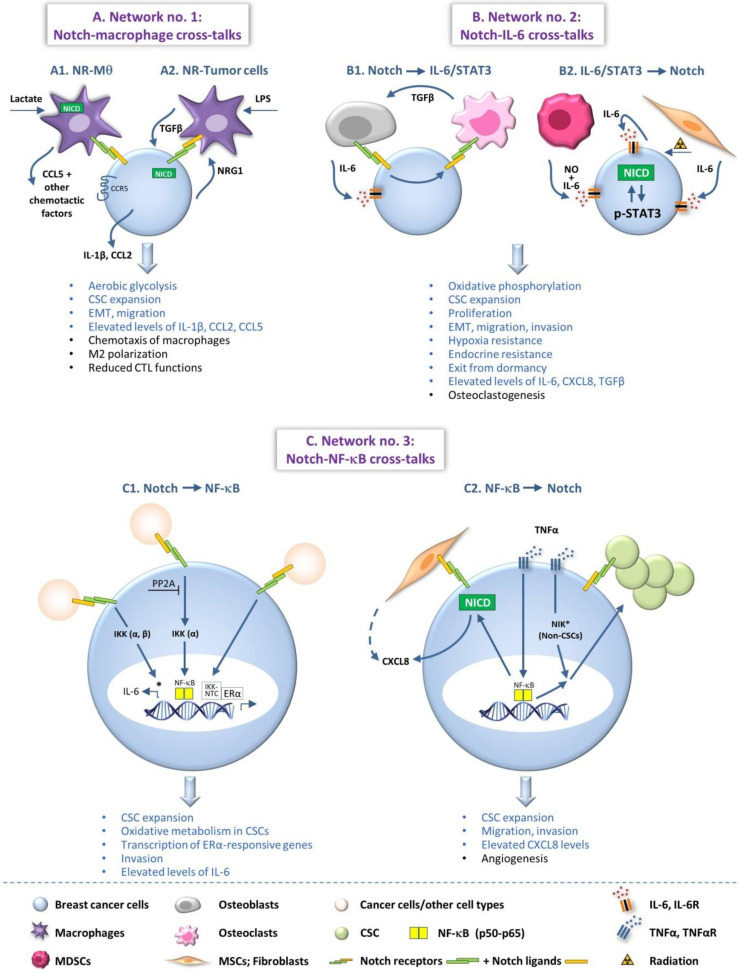
Notch-inflammation networks that promote breast cancer progression. The Figure demonstrates three major networks that are established between members of the Notch pathway and inflammatory elements in BC. For the sake of simplicity, not all processes and not all mechanistic details that were described in the different studies are demonstrated in the Figure. When several processes that were identified in different publications are illustrated in the same cell/setting, there is no intention to imply that they take place simultaneously. In the text, the readers are referred to the relevant parts that are demonstrated in the Figure. Three Notch-inflammation networks are demonstrated in the Figure: (**A**) Network no. 1—Cross-talks between the Notch pathway and macrophages, as key representatives of myeloid cells that contribute to cancer-related inflammation; (**A1**) Effects of tumor-induced activation of Notch signaling in macrophages; (**A2**) Effects of macrophage-induced activation of Notch signaling in the tumor cells. (**B**) Network no. 2—Cross-talks between the Notch pathway and IL-6—a major pro-inflammatory cytokine that is one of many pro-inflammatory factors prevailing in breast tumors and promoting disease progression—and its downstream pathways (STAT3); (**B1**) Effects of Notch activation on IL-6/STAT3 signaling; (**B2**) Effects of IL-6/STAT3 signaling on Notch activation in the tumor cells. (**C**) Network no. 3—Cross-talks between the Notch pathway and the NF-κB transcription factors, which have prominent roles in inflammatory processes and mediate pro-inflammatory/pro-metastatic effects in many malignancies, including BC; (**C1**) Effects of Notch signaling through activation of the NF-κB pathway, on breast tumor cells; (**C2**) Effects of NF-κB-mediated effects through Notch activation on breast cancer cells. *, aspects concerning the activation of non-classical NF-κB pathways. In the texts describing the pro-tumor consequences of the Notch-inflammation networks, the blue text signifies pro-malignancy effects potentiated in the tumor cells and the black text illustrates tumor-promoting activities at the TME. Please note that factors released by the cancer cells can also affect the characteristics of the TME. CSCs, cancer stem cells; CTLs, cytotoxic T cells; EMT, epithelial-to-mesenchymal transition; ERα, estrogen receptor α; IKK, IκB kinase; IL-6, interleukin 6; IL-6R, IL-6 receptor; LPS, lipopolysaccharide; MDSCs, myeloid-derived suppressor cells; MSCs, mesenchymal stem cells; Mθ, macrophages; NICD, Notch intracellular domain; NIK, NF-κB inducing kinase; NO, nitric oxide; NR, Notch receptors; NRG1, Neuregulin 1; NTC, Notch–CSL–MAML1 transcriptional complexes; TGFβ, transforming growth factor β; TNFα, tumor necrosis factor α; TNFαR, TNFα receptor.

This association of Jag1 expression with macrophages suggests that members of the Notch family may be functionally connected to pro-tumor macrophage activities in BC. Indeed, several studies indicated that the expression of Notch receptors by macrophages played important roles in regulating the pro-malignancy functions of BC cells (Figure 1(A1)). In other cases, BC cells were those that expressed Notch receptors (Figure 1(A2)), and eventually vicious cycles of interactions between macrophages and BC cells through Notch receptors and their ligands were reported, as described below.

An example for tumor interactions with Notch-expressing macrophages was identified when lactate-treated macrophages demonstrated elevated release of the chemokine CCL5, induced through Notch1 signaling; then, the chemokine induced the activation of its corresponding receptor CCR5 in the cancer cells. This interaction has led to increased aerobic glycolysis in the tumor cells, which also acquired elevated epithelial-to-mesenchymal properties and migratory activities [85] (Figure 1(A1)). Additional evidence for Notch-regulated networks was revealed in BC when in long-term endocrine depleted luminal-A BC cells (LTED) the expression of Jag1 was elevated, and macrophages that grew in culture with such LTED cells acquired an M2 phenotype, in a Notch-dependent manner. The resulting M2 macrophages released factors that elevated the migration of the tumor cells, thus revealing an interactive loop between the cancer cells and macrophages via members of the Notch pathway [83] (Figure 1(A1)).

In parallel, it was found that the terminal differentiation of macrophages to TAMs—that did not demonstrate the alternatively activated phenotype of M2 macrophages—depended on recombination signal-binding protein for immunoglobulin kappa J region (RBPJ) [86], known to act together with Notch intracellular domain (NICD) in gene expression regulation [87]; in turn, these RBPJ-expressing TAMs were found to down-regulate the levels of granzyme-expressing CD8+ T cells and to elevate tumor burden in a BC model [86].

As indicated above, other studies demonstrated that the reciprocal mode of activation can also exist, in which the tumor cells are those expressing Notch receptors. For example, in a study of basal-like BC cells it was found that lipopolysaccharide (LPS) treatment of macrophages induced the expression of Jag1 in these cells; based on other findings provided in that study, the authors suggested that through such Jag1 up-regulation the macrophages could have led to Notch activation in cancer stem cells (CSCs) and then to their expansion [88] (Figure 1(A2)). In another study, it was found that TNBC cells expressed neuregulin 1 (NRG1) that activated macrophage-expressed ErbB3, leading to increased expression of Jag1 by the macrophages; then, when the two cells interacted in co-culture, Jag1 was necessary for induction of tumor cell trans-endothelial migration (presumably by binding to cancer cell-expressed Notch) [89].

Another illustration to a situation in which Notch receptor-expressing BC cells and macrophages cross-talk with one another, was found in a recent study demonstrating that TAM-derived transforming growth factor β1 (TGFβ1) has activated the Notch1 pathway in basal-like BC cells, giving rise to increased production of IL-1β and of the monocyte chemoattractant CCL2 in the presence of macrophages [90]; as a result, the recruitment of macrophages to the tumor area was increased, and has further facilitated the TGFβ-mediated activation of the tumor cells by macrophages through Notch (Figure 1(A2)). In this case, both Notch3 and Jag1 were involved in the process and their inhibition gave rise to smaller tumors, accompanied by reduced IL-1β and CCL2 levels in the tumors and lower macrophage presence [90].

Overall, the above findings illustrate the interactions between BC cells and macrophages that are mediated by Notch-related pathways, and demonstrate how such processes can generate vicious cycles that strongly promote disease course. In addition, the Notch pathway can dictate the phenotype of leukocytes that infiltrate the tumor site, for example by regulating the expression of chemokines that promote the presence of TAMs at the tumor setting, by that strongly affecting the malignancy cascade in BC.

## 3. Network No. 2: Cross-Talks of the Notch Pathway with Pro-Inflammatory Soluble Mediators

The TME is enriched not only with inflammatory cells but also with a large number of pro-inflammatory soluble mediators. Many of these factors were linked to a less favorable disease course in cancer, and play causative roles in promoting tumor progression. Specifically, pro-inflammatory cytokines and inflammatory chemokines were reported to have a strong tumor-supporting impact on cancer development and metastasis, as illustrated by many studies of BC and other cancer types as well (e.g., [45,74,75,76,77,78,79,91,92,93,94,95,96]).

In parallel, different studies indicated that the pro-inflammatory mediators TNFα and IL-1β, as well as the inflammatory chemokines CXCL8 (IL-8), CCL2 (MCP-1) and CCL5 (RANTES)—that are major contributors to BC progression [45,74,75,76,77,78,79,93,94,95,96]—were connected to Notch activities in BC. For example, evidence from our studies indicated that co-culturing of TNBC cells with mesenchymal stromal cells (MSCs) or cancer-associated fibroblasts (CAFs) in the presence of TNFα or IL-1β stimulation has given rise to contact-dependent increase in CXCL8, CCL2 and CCL5 levels in the co-cultures. Under these conditions, it was Notch1 activation—mainly in the cancer cells but also to some degree in the MSCs—that has led to CXCL8 up-regulation (to be described in more detail below; see also Figure 1(C2)). Then, CXCL8 has significantly contributed to elevated tumor cell migration and invasion, and also to increased angiogenesis [97,98]. Eventually, TNBC cells that formed physical contacts with MSCs in the presence of TNFα stimulation gave rise to increased incidence of lung metastasis [97]. Other research works in the field also indicated that these same inflammatory factors stand in the basis of Notch interplays that contribute to BC progression [85,88,90,99,100,101,102].

With these findings in mind, it is interesting to note that another pro-inflammatory cytokine, IL-6 has been highly implicated in tumor-promoting processes [79,91,92] and in Notch-related pathways in BC. Here, two main lines of evidence were obtained: the first is that Notch pathway activities have given rise to tumor-promoting functions in BC via IL-6 and STAT3 activation (Figure 1(B1)), and the second is that IL-6 stimulation/STAT3 activation has led through Notch family members to pro-metastatic activities in BC (Figure 1(B2)).

In the first line of research, the ability of Notch family members to activate IL-6/STAT3-mediated pathways was reported in a study that addressed resistance of luminal-A BC cells to tamoxifen. In this research, it was found that Notch4 activated STAT3 and that this pathway has induced epithelial-to-mesenchymal transition (EMT) in tamoxifen-resistant tumor cells [103]. In this context, it is interesting to note that complex interactions between the Notch and IL-6 pathways were reported in another study. In this case, inhibition of Notch3 has given rise to elevated tumor growth and to increased IL-6 expression by the tumor cells, which then caused CSC expansion. This effect of Notch3 inhibition was abrogated by IL-6R antagonist in animal studies and reduced the generation of CSCs. When general inhibition of the Notch pathway was introduced in animal studies by γ-secretase inhibitors (GSI), the addition of IL-6R antagonist has potentiated the ability of GSI to reduce the frequency of CSCs in tumors [104].

In another study that exemplifies the roles of Notch pathway in inducing IL-6/STAT3 activation in BC tumorigenesis, it was found that Jag1–IL-6 interactions acted on the TME and have led to increased bone metastasis in a TNBC model (Figure 1(B1)). This study demonstrated that Jag1 expression by the tumor cells mediated their interactions with osteoblasts, promoting their ability to release IL-6 that elevated tumor cell proliferation. In addition, it was found that activated Jag1 mediated interactions of the tumor cells with osteoclasts [105]. In turn, osteoclasts have induced bone osteolysis and facilitated bone metastasis, accompanied by increased release of TGFβ; TGFβ-SMAD signaling has then further supported Jag1/IL-6-mediated interactions between the osteoblasts and the cancer cells, leading to a positive feedback loop that further promoted the metastatic process in bones [105] (Figure 1(B1)).

In parallel, the reciprocal direction of IL-6/STAT3-mediated induction of Notch activation was also reported. In this context, it was demonstrated that MDSCs elevated tumor growth and stemness in BC, and also produced IL-6 and nitric oxide (NO). In turn, IL-6 has induced STAT3 activation and NO has given rise to increased Notch activation in tumor cells (Figure 1(B2)). Of interest was the fact that Notch activities have led to persistent activation of STAT3 following IL-6 stimulation, and the cooperative activities of STAT3 and the Notch pathway resulted in increased generation of CSCs [67]. In a similar manner, autocrine IL-6-driven STAT3 activation has given rise to Notch3/Jag1 activation that sustained the enrichment of CSCs, and through Notch3 activation also has led to hypoxia resistance and increased invasion of BC cells [106] (Figure 1(B2)). In a parallel study, IL-6 was directly connected to BC stemness by inducing the activation of integrin-linked kinase (ILK), which has led to Notch1 activation and to assembly of functional γ-secretase complexes localization in caveolae, then giving rise to elevated CSC expansion [107].

Interestingly, in regards to Notch-inflammation regulation of stemness in BC, a recent study integrated mathematical modeling to reveal the underlying principles of experimentally-observed intra-tumor heterogeneity of different subsets of CSCs. This computational model predicted that due to spatial gradients of EMT-inducing signals such as TGFβ together with Notch-Jag1 activation, CSCs exhibit varying EMT phenotypes depending on their localization at the tumor mass, from the invasive edge to the core. Moreover, this study demonstrated that inflammatory cytokines such as IL-6, enable a subpopulation of cancer cells to display a hybrid epithelial/mesenchymal phenotype by enhancing Notch-Jag1 signaling and increasing the CSC population [108].

In addition to the IL-6-Notch interactions that promoted stemness, this axis was also found to up-regulate tumor cell motility in BC. Fractionated radiation of BC cells has led to increased production of IL-6 and CXCL8, and inhibition of each of these factors has given rise to reduced tumor cell migration and invasion (Figure 1(B2)); here, it was also found that IL-6 acted through JAK/STAT3 to activate Notch2-Jag1/DLL4 interactions that elevated EMT characteristics in BC cells [109] (Figure 1(B2)). In another research, it was demonstrated that IL-6 derived from senescent fibroblasts has led through STAT3 activation to Notch3 and Jag1 activation [110] (Figure 1(B2)). In addition, IL-6 activities in BC were found to be mediated through activation of the bromodomain and extraterminal (BET) protein BRD4 that was required for Notch1-mediated pro-tumor activities [25]. In these cases, the activation of these members of the Notch pathway increased tumor cell migration, invasion and proliferation [25,110] (Figure 1(B2)).

Complementing these levels of IL-6/STAT3-driven pro-tumor activities in BC is the aspect of endocrine resistance. A recent study demonstrated that ER-expressing BC cells acquired endocrine resistance that was accompanied by elevated oxidative phosphorylation, in a process that depended on IL-6/STAT3-driven activation of Notch3. Eventually, this process stood in the basis of tumor cell self-renewal of CD133-high/CD44-low cells that has given rise to exit from dormancy and metastatic recurrence of ER-low BC cells that were resistant to endocrine therapy [111].

To conclude, the above investigations indicate that Notch-IL-6 interplays stand in the center of tumor progression in BC, where the interactions could be reciprocal, depending on the cell system and on the parameters that were addressed. Here, Notch family members could lead to IL-6/STAT3 activation, and in other cases the IL-6/STAT3 pathway has given rise to Notch activation, eventually promoting a diversity of tumor-promoting processes in BC cells.

## 4. Network No. 3: Cross-Talks of the Notch Pathway with Transcription Factors that Promote Inflammatory Processes

Transcriptional regulation of cancer-related inflammatory processes is complex and orchestrated by many signaling pathways and transcription factors such as STAT3, AP-1 and NF-κB. The findings summarized above on networks established between the Notch pathway and IL-6 portrayed the tight interplay that exists between Notch receptors/ligands with STAT3 in BC; several lines of evidence also addressed the interactions of Notch family members with JNK/AP-1 in BC [112,113].

In parallel, growing interest has been demonstrated in cross-talks that may be established between the Notch pathway and the NF-κB pathway. Here, the different studies revealed interactions of Notch with the classical (canonical) NF-κB pathway in which heterodimers of IκB kinase α (IKKα) and IKKβ lead to activation of p50 and p65 (RELA), typically induced by pro-inflammatory cytokines such as TNFα and IL-1β and infection-related signals. Other reports indicated that Notch also cross-talks with the non-classical (alternative, non-canonical) NF-κB pathway, where the players are mainly NF-κB inducing kinase (NIK), homodimers of IKKα and the activation of p52 and RELB; in this case, activation is induced by divergent stimulators, including members of the TNF family members, such as lymphotoxin [114,115].

To date, many reports have described a bi-directional regulation of Notch and NF-κB through different, context-dependent mechanisms and their diverse roles in physiological and in pathological conditions (representative reviews: [116,117,118,119]). Similarly, in cancer, efforts were devoted to deciphering Notch-NF-κB interactions and the way they affect the malignant behaviors of tumor cells and of the TME [114,115,120,121]. Cross-talks between the Notch pathway and the NF-κB-mediated route—primarily of the classical mode—are highly relevant for BC progression. In this malignancy, members of the Notch pathway play prominent roles in regulating disease course (as was described above) and in parallel elevated activation of NF-κB drives BC tumor development and progression at many different levels [114,120,121].

Indeed, it was found that the Notch and NF-κB pathways promote BC progression by regulating each other’s activities, at many levels. Here, again, reciprocal modes of activation were revealed: one mode in which members of the Notch pathway have activated the NF-κB route (Figure 1(C1)) and a second mode in which NF-κB activation has given rise to stimulation of the Notch pathway (Figure 1(C2)).

One of the indications to possible Notch1-mediated increase in malignant features of BC cells through p65 activation was provided by a study demonstrating that Notch1 activation in TNBC cells increased the proliferation, adhesion, invasion and motility of the tumor cells. In parallel, Notch1 induced accumulation of p65 in the nucleus, elevated IκBα degradation and up-regulated the formation of NF-κB-DNA complexes, suggesting that Notch1 induced tumor-promoting activities by activating NF-κB [122]. In a follow-up study, direct evidence to such a scenario was revealed in motility-related aspects, when the authors have shown that Notch1-induced invasion of the cancer cells depended on NF-κB activation. Moreover, the study has shown that Notch1-induced NF-κB activation was governed by the PI3K/AKT pathway and IKK, and was negatively regulated by PP2A [123] (Figure 1(C1)). In another report, where Notch1 was found to induce pro-tumor activities in luminal-A BC cells and in the non-transformed breast epithelial cells in culture, Notch1 was also found to induce p65 activation [101].

Complementing these findings, another study revealed that the Notch pathway up-regulated NF-κB activation, mitochondrial metabolism and CSCs in TNBC cells. Here, it was found that activation of Notch1 by Jag1 promoted cellular bioenergetics via IKKα-mediated activation of AKT in TNBC cells; In parallel, Notch1 activation in TNBC cells by Jag-expressing fibroblasts has promoted the recruitment of p50, p65 and IKKα to cIAP-2 promoter [124] (Figure 1(C1)). In addition, GSI treatment in combination with IKK or AKT inhibitors significantly abrogated the growth of mammospheres that were generated from TNBC cell lines and from flow-sorted CD44+/CD24-low or CD90-high CSCs. Eventually, it was found that mammospheres had increased oxidative metabolism that was sensitive to inhibition of the Notch, IKK and AKT pathways. Overall, these data indicate that Jag1-Notch1 acted through IKKα to trigger two parallel and potentially interacting pathways: mTORC2-dependent AKT activation that increased mitochondrial metabolism and a p50-p65-mediated pathway that triggered transcriptional activation [124] (Figure 1(C1)).

Key roles of IKKα in mediating Notch signaling were also revealed in luminal-A BC cells, where IKKα–Notch interactions promoted gene transcription by ERα. This study provided evidence to a complex network of interactions, in which Jag1-induced Notch1 activation was followed by the recruitment of IKKα to Notch–CSL–Mastermind-like 1 (MAML1) transcriptional complexes (NTCs), accompanied by ERα binding and transcription of relevant genes; Notch1-induced p300 recruitment by MAML1 could also give rise to transcription of ERα-responsive genes in the absence of estrogen [125] (Figure 1(C1)).

In addition to controlling the classical NF-κB pathway, it was found that Notch could elevate pro-inflammatory characteristics in BC through non-classical NF-κB mechanisms [126]. In this study, it was demonstrated that Notch-mediated induction of IL-6 was inhibited by siRNA to IKKα or IKKβ, but was independent of p50 of the classical NF-κB route; moreover, Notch activation did not affect the activation levels of p65 [126] (Figure 1(C1)). Therefore, this research has demonstrated that not only classical but also non-classical NF-κB mechanisms are activated by the Notch pathway in BC.

The above findings demonstrated that the Notch pathway plays important roles in un-regulating the activation of NF-κB-related mechanisms, which eventually have led to pro-tumor effects in BC. As mentioned above, evidence for the reciprocal axis was also provided, indicating that the NF-κB pathway controlled Notch-mediated signaling (Figure 1(C2)). Here, our recent publications aforementioned [97,98] demonstrated that TNFα stimulation of direct co-cultures of TNBC cells with MSCs has given rise to elevated migration and invasion of the tumor cells and to increased release of angiogenic factors, in a process that was CXCL8-dependent. The TNFα-induced increase of tumor cell motility and CXCL8 production by contact TNBC co-cultures with MSCs were inhibited by GSI, and direct involvement of Notch1 was demonstrated in up-regulating CXCL8 production [97,98]. In analyses of the molecular mechanisms we found that following TNFα stimulation of such co-cultures, p65 was activated in both cell types but more so in the cancer cells; moreover, p65 activation mainly in TNBC cells, but also in the MSCs, up-regulated the expression and activation of Notch1 in the tumor cells, which then promoted CXCL8 production primarily in the cancer cells [98] (Figure 1(C2)). Overall, our data demonstrated that p65-induced Notch1 activation was required for the contact-dependent induction of CXCL8 in the tumor-stroma-inflammation network, promoting malignancy-related function in TNBC cells.

In parallel, in a study mentioned above [88] it was found that NF-κB-induced up-regulation of Jag1 expression in non-CSCs has led to activation of the Notch pathway in CSCs and to expansion of this sub-population. In this investigation, NF-κB was activated by TNFα or in a non-classical manner by NIK, leading through Jag1 to activation of Notch signaling in the CSC sub-population (Figure 1(C2)). Moreover, BC clinical data and additional in vitro analyses suggested that the NF-κB-Jag1 axis was unique to the basal-like subtype and had specific role in the maintenance of the CSCs [88]. The findings of this study also suggested that Jag1-expressing macrophages, fibroblasts and other cells of the breast TME, may contribute to the self-renewal of CSCs in an NF-κB-Notch-dependent manner [88].

The findings described above provide evidence to complex interactive relationships of the Notch pathway with transcription factors that are typically involved in inflammatory processes, which may act in bi-directional and cooperative manners. Together with the previous sections, which exemplified other levels of Notch-inflammation cross-talks, the Notch-NF-κB network emphasizes the profound impact of combined Notch-inflammation regulatory networks on BC progression.

## 5. Discussion

Notch receptors and their ligands stand in the center of many developmental and pathological processes, in which they exert their impacts by mediating cell-to-cell contacts. This highly conserved pathway also strongly affects tumor progression by mediating the signal flow from external elements to transcription regulation. An increasing body of evidence indicates that the TME, with its pro-inflammatory elements, is strongly connected to Notch pathway regulation and activities. Furthermore, members of the Notch family are connected to transcription factors that are typically involved in regulation of inflammatory conditions.

In this review article, we chose to focus on BC in order to exemplify the interplay between Notch family members and inflammation. In this malignancy, both partners were shown to play key roles in promoting disease course, and recent studies indicate that multiple levels of interactions between them take place and promote tumor progression. In this context, we described the interplays of the Notch pathway with three inflammatory elements: (1) Myeloid inflammatory cells, mainly macrophages; (2) Pro-inflammatory soluble mediators, primarily IL-6 and its signaling pathway; and (3) Transcription factors that integrate pro-inflammatory signals, with major emphasis on NF-κB. These three networks indicated that Notch1 and Jag1 were the key family members that participated in Notch-inflammation cross-talks, with observations pointing at other partners - such as Notch2, Notch3, Notch4 and DLL4 - as important potential regulators in these settings, as well.

The research works summarized above have indicated that Notch-inflammation interplays strongly promote the malignancy phenotype, in vitro and/or in vivo, by advancing pro-tumor processes that take place primarily in the malignant cells but also at the TME. Specifically in the cancer cells, many different levels were positively affected by the Notch-inflammation networks, such as: increase in metabolic processes; CSC expansion; elevated tumor cell proliferation; increased EMT, migration and invasion; higher resistance to hypoxia and endocrine treatments, and transcription of ERα-responsive genes; and elevated release of pro-inflammatory /metastasis-promoting factors such as IL-1β, IL-6, CCL2, CXCL8 and TGFβ. Obviously, the release of such soluble mediators can mediate the impacts of cancer cells on their TME. In parallel, Notch-inflammatory interactions could lead at the TME to increased levels of TAMs that have tumor-promoting activities; they could also dictate the phenotype of the macrophages at the tumor site, induce immune-suppression, mediate tumor-stroma interactions that promote malignancy, enhance angiogenesis and elevate metastasis by increasing bone osteolysis.

The above findings revealed shared characteristics between the three Notch-inflammation networks. First, the tumor-promoting activities of Notch-inflammation interactions were noted in several subtypes of BC, and were not limited to luminal-A or TNBC cells. Thus, it is highly plausible that Notch-inflammation interactions actually have a general tumor-promoting impact in BC, as may be the case in other malignancies as well. Therefore, Notch-inflammation networks may be considered as a therapy target shared by many cancer types. Yet, as tumors demonstrate a high level of inter-tumor and intra-tumor heterogeneity, the impacts and the specific participants of Notch-inflammation networks may depend on disease types and subtypes and may also reflect the diversity in the research systems and tools used. This variability calls upon extensive research that will identify the precise mechanisms that connect the Notch pathway and inflammatory elements in different types of cancer.

Second, in all three networks described in this review article, the Notch-inflammation interplay was reciprocal, exemplified by the ability of the Notch pathway to induce pro-inflammatory conditions and vice versa. As much as Notch family members were found to promote tumor-supporting functions of macrophages, macrophages affected pro-metastatic activities in BC cells through the Notch pathway. In similarity, Notch activation could stimulate IL-6-induced pathways, and in parallel the reciprocal direction was active, both leading to pro-tumor activities in BC. By the same token, Notch signaling pathways often promoted tumor-related functions by activating the NF-κB pathway, and Notch activation by the NF-κB pathway was also taking place in the BC setting.

These reciprocal relationships between Notch and inflammation, exerted through many different mechanisms and generally leading to pro-tumor activities in different BC subtypes, illustrate the strength and importance of the interactive Notch-inflammation network. Thus, these findings set the Notch pathway and inflammatory elements as therapeutic targets that should be pursued simultaneously in BC treatment, and possibly in other cancer types as well.

In recent years, different GSIs demonstrated encouraging anti-tumor potential in pre-clinical studies of several cancer types including of the breast (reviewed in [8,14,127]). Yet, many of the clinical trials that have evaluated GSIs for their efficacy as mono-therapies have led to unsatisfactory results or were terminated due to low tolerability and/or limited dose-dependent anti-tumor activity. As single treatment, GSIs may present several potential disadvantages. First, GSIs fail to distinguish between the different Notch receptors, some of which may have context- and tumor-dependent opposing roles. Furthermore, as γ-secretase cleaves additional targets, GSI treatment might result in off-target effects and unselective inhibition of other signaling pathways. Additional limitation of long-term GSI treatment is the gastrointestinal toxicity since Notch signaling is required for maintaining the normal function of the intestinal epithelium [128].

To address the specificity concerns of pan-Notch pathway blockade in the clinic, monoclonal antibodies (mAbs) were and are currently developed as a new therapeutic strategy for a specific inhibition of Notch signaling. For instance, anti-DLL4 mAbs (Demcizumab) and mAbs targeting Notch 2/3 (Tarextumab) are being evaluated in pre-clinical and in early phase clinical settings for their safety and therapeutic effects in solid tumors including of the breast, demonstrating initial evidence for anti-tumor activity and/or disease stabilization [8,129,130,131]. However, similarly to GSIs, clinical use of one specific mAb as a single agent most likely will not eliminate the potentially harmful side-effects of Notch pathway inhibition in non-cancerous tissues; in fact, dose-limiting toxicities have been reported in phase I studies of such mAbs [129,130,132].

Data presented in this review on the central roles of each of the partners alone and more so, of combined Notch-inflammation networks, strongly suggests that in future clinical studies it would be beneficial to aim at combination therapies targeting these two axes together as a rational strategy for BC. This option is especially attractive when the toxicity of GSIs is considered, raising the possibility that in the presence of inflammation inhibitors, the doses of GSI or other Notch inhibitors (e.g., Abs) could be reduced. Indeed, some of inflammation inhibitors are already in clinical use. For example, mAbs against the IL-6 receptor are in clinical use for the treatment of rheumatoid arthritis [133]. TNFα and IL-1β inhibitors are in clinical use for inflammatory diseases and autoimmune disorders [134,135,136] and were demonstrated to inhibit the aggressiveness phenotype of BC cells (as cited above). Under conditions of simultaneous inhibition of Notch-inflammation, it is possible that more effective and safer treatment modalities could be offered to BC patients and to individuals suffering from other malignancies alike.
